# Comparison of Marker‐Based RSA and CT‐RSA for Analyzing Micromotions After Distal Radius Osteotomy: A 1‐Year Retrospective Study of 24 Patients

**DOI:** 10.1002/jor.26031

**Published:** 2024-12-27

**Authors:** Vasileios Angelomenos, Olof Sandberg, Bita Shareghi, Michael Ullman

**Affiliations:** ^1^ Department of Orthopedics, Institute of Clinical Sciences, Sahlgrenska Academy University of Gothenburg Gothenburg Sweden; ^2^ Department of Hand Surgery Sahlgrenska University Hospital Gothenburg Sweden; ^3^ Sectra Linköping Sweden; ^4^ Department of Orthopedics Sahlgrenska University Hospital Gothenburg Sweden

**Keywords:** computed tomography, CT‐based, distal radius, micromotions, radiostereometric analysis

## Abstract

Radiostereometric Analysis (RSA) is the most accurate method for determining early micromotions of orthopedic implants. Computed Tomography Radiostereometric Analysis (CT‐RSA) is a method that can be used to determine implant and bone micromovements using low‐dose CT scans. This study aimed to evaluate the reliability of the CT‐RSA method in measuring the interfragmental mobility in patients who have undergone a correction osteotomy due to a malunited distal radius fracture. Twenty‐four patients were included and operated with a radiolucent volar plate. Markers were embedded in the plate and bone. RSA and CT examinations were obtained postoperatively up to 1‐year postoperative. Micromovements of the distal radius segment relative to the proximal were compared between the methods with paired analysis and Bland–Altman plots. The limits of clinical significance were: dorsal/volar tilt < 10°, radial shortening < 5 mm, radial inclination ≥ 15°, and radial shift < 5 mm. For the dorsal/volar tilt, the paired analysis between the two methods, showed a mean difference (95% CI) of −0.06° (−0.67 to 0.55), for radial compression‐0.04 mm (−0.09 to 0.01), for radial inclination 0.21° (−0.06 to 0.48), and for radial shift −0.07 mm (−0.21 to 0.07). The paired analysis for micromotions showed that the thresholds of clinical significance are excluded from the difference's 95% CI. The Bland–Altman plots showed comparable results up to 1 year, considering clinically relevant thresholds. In conclusion, the CT‐RSA method is comparable to that of marker‐based RSA in measuring micromotions after wrist osteotomy, as the differences between the methods are not clinically significant.

## 1 Introduction

Correction osteotomy is a well‐established method in the treatment of malunion of a distal radius fracture that has been treated conservatively or operatively [1]. Despite this fact, research is limited on postoperative inter‐fragmental mobility as well as relative to the osteotomy plate.

Since its introduction by Selvik in 1974 [2], marker‐based radiostereometric analysis (RSA) has been the gold standard in measuring micromotions of orthopedic implants of the hip, knee, and shoulder [3]. However, there are only a handful of studies utilizing RSA in the analysis of micromotion in the wrist after osteosynthesis due to a fracture or osteotomy of the wrist [4, 5, 6, 7, 8, 9]. There are several reasons for this: marker‐based RSA requires the implantation of tantalum markers in both the implant and bone segments to be analyzed. In addition, the wider apart the distribution of the markers is within the different moving bodies, the higher the reliability of the measurements of micromotion [10, 11] as it translates to a lower condition number (CN). However, the wrist is an anatomically smaller region with complex soft tissue anatomy and, therefore, it is rather inconvenient to implant tantalum markers wide apart and well distributed, especially within the distal radius fragments, making the RSA analysis challenging. This problem was partially solved with the introduction of model‐based RSA [12, 13], something that has been previously used in measuring the micromotion of wrist implants [14].

The principles of CT‐RSA have been investigated for the last two decades [15, 16, 17]. It uses a minimum of two low‐dose CT scans, with or without the use of implanted markers [18], at predefined time points and utilizing thresholds to identify the bone and/or the implant. The reference and moving bodies are registered and the relative movements are calculated. Compared to marker‐based RSA, image acquisition might be easier, and the precision in measuring micro‐movement is shown in previous studies to be comparable to that of RSA [18, 19]. These facts have led researchers to propose CT‐based methods as an alternative to marker‐based RSA in evaluating implant migration and rotation in major joints [15, 19].

**Figure 1:**
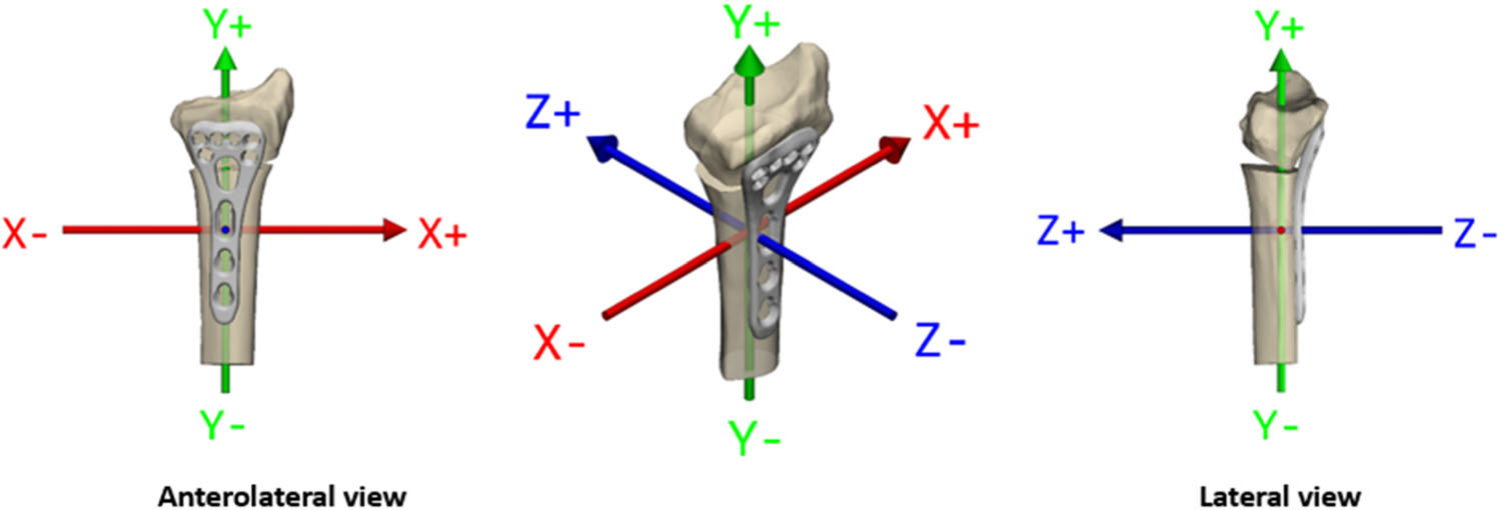
Axes of interest in distal radius displacement. The dorsal/volar tilt corresponds to rotations around the *x‐*axis, radial inclination to rotations around the *z‐*axis, axial compression to translations along the *y‐*axis, and radial shift to translations along the *x‐*axis.

The purpose of this study was to evaluate the reliability of CT‐RSA in measuring inter‐fragmental mobility compared to marker‐based RSA in patients undergoing a correction osteotomy due to a malunited distal radius fracture. This was done under as similar measurement conditions as possible. The primary outcome measure in this study was set as the dorsal/volar tilt of the distal radius bone segment which equals to rotation around the *x*‐axis. The secondary outcome measures were the axial shortening of the osteotomy gap which equals to translation along the *y*‐axis, change in radial inclination which equals to rotations around the *z*‐axis, and radial shift which equals to translation along the *x*‐axis (Figure 1). Based on previous research, as explained in the discussion, the limits of acceptable distal radius displacement were set to: dorsal/volar tilt < 10° (rotation in the *x*‐axis), radial shortening < 5 mm (translation in the *y*‐axis), radial inclination ≥ 15° (rotation in the *z*‐axis), and radial shift < 5 mm (translation in the *x*‐axis).

Our hypothesis was that the differences in measurements of micromotion with the two methods are not clinically significant. Furthermore, we quantified the differences in CT‐RSA measurements when the RSA coordinate system was used versus the actual position of each patient's forearm in the measurement.

## 2 Materials and Methods

**Figure 2:**
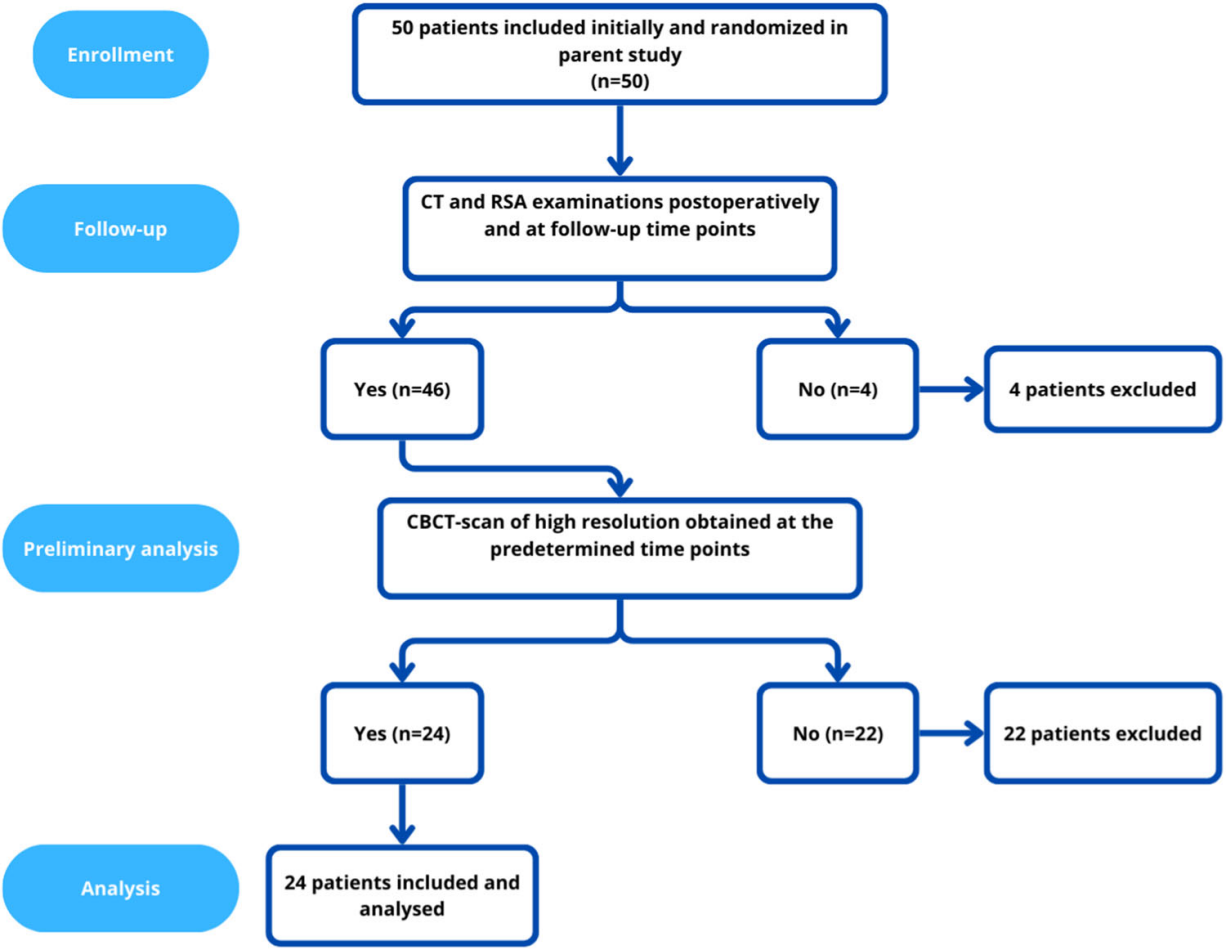
Flowchart showing inclusion of patients in the current study.

This is a retrospective cohort study with a Level III of evidence. Twenty‐four patients were included in this study, 6 males and 18 females with a median age of 63 (15–78). The inclusion criteria were diagnosis of a symptomatic distal radius malunion after conservatively treated distal radius fracture, suitable for extra‐articular distal radius osteotomy. All patients were operated at the Department of Orthopedics at Sahlgrenska University Hospital, Mölndal, Sweden by the same surgeon (M.U.) between 2014 and 2018. All patients were part of a larger RSA study at the same institute and were included in this study only if they had complete RSA examinations (that fulfilled the criteria for RSA analysis) and CT scans at two occasions, directly postoperatively and at a time point when the osteotomy was considered clinically and radiologically healed. Fifteen patients were, thus, examined with follow‐up marker‐based RSA and CT‐RSA 12 months postoperatively, five patients after 6 months, and four patients 3 months postoperatively. Please see Figure 2 regarding patient inclusion workflow. All patients gave informed consent for this study. This study followed the STARD guidelines, and the corresponding checklist is available as Supporting Information.

### 2.1 Surgical Technique

A standard modified Henry approach was used volarly to access the volar distal radius. A small longitudinal dorsal incision of 35–40 mm was made, the third extensor compartment was released, and the second and fourth extensor compartment were lifted subperiosteally to expose the dorsal radius. Following established RSA guidelines [20], to enable marker‐based RSA analysis six to seven Tantalum markers (RSA Biomedical, Umeå, Sweden), with a diameter of 1.0 mm, were inserted volarly in the proximal and distal bone segments using a designated inserter (RSA Injector, RSA Biomedical, Umeå, Sweden), before the osteotomy was performed with a power saw. The desired correction was established with a distractor, and temporarily fixed with a preformed plastic wedge, inserted in the osteotomy void. The osteotomy was stabilized by a volar DiPhos R radiolucent plate (Lima Corporate, Udine, Italy), made of carbon fibers and PEEK (PolyEtherEtherKetone‐polymer) fixed with titanium screws. The plate was manufactured with embedded tantalum markers of 0.8 mm diameter, to make it visible on radiographs. In addition, at surgery, as a part of a larger study, the patients were randomized to be operated with or without the injection of hydroxyapatite bone substitute in the osteotomy void (Hydroset, Stryker Corp, Kalamazoo, MI, USA). This difference in treatment was not a factor in the present study, as the purpose was to compare the reliability of the two methods of micromotion analysis.

### 2.2 RSA

All marker‐based RSA measurements and analyses were performed using UmRSA Digital Measure and UmRSA Analysis software version 7.0 (RSA Biomedical, Umeå, Sweden). The software was used to calculate the rotation and translation around and along the three orthogonal axes (*x*, *y*, and *z*) (Figure 1). RSA examinations were done postoperatively and up to 1 year postoperative. On the methods described by ISO standard ISO16087:2013 [20] on precision regarding marker‐based RSA it is stated that a “minimum of 25% of the cases evaluated or 15 cases (whichever of the two represents the largest number of patients) shall be evaluated with double examinations.” To calculate the precision of the RSA method, 19 double RSA examinations were done after the osteotomy was considered clinically and radiologically healed and the differences in the measured translation and rotation between them were measured. Between the double RSA examinations, done a few minutes apart, the patient's hand was repositioned without changing the RSA set‐up; that is, the position of the calibration cage and x‐ray tubes [21]. We assumed that no true motion of the plate or radius shaft or distal radius segment had occurred between the two consecutive RSA examinations.

All examinations were conducted using an Adora radiographic system (NRT‐Nordisk Røntgen Teknik A/S, Hasselager, Denmark). The uniplanar technique with the RSA calibration cage under the examination table was utilized (cage 77, RSA Biomedical, Umeå, Sweden). All RSA analyses were done by a single biomedicine scientist with a long clinical and research experience in RSA. For larger bones and implants, the upper limit of 150 for the CN has been suggested [10]. For small bones, though, such as in the wrist and hand higher CNs should be expected [11, 22]. In the RSA software, the rigid body error describes the stability of the bone markers. An upper limit of ridig body error of 0.35 mm has been previously suggested [10]. In the current study, we followed these guidelines and evaluation of radiostereographs was performed only if three or more tantalum markers in the segments distal as well as proximal to the osteotomy could be identified with a scatter corresponding to a CN less or equal to 200 and a rigid body error less or equal than 0.35 mm.

### 2.3 Computed Tomography Radiostereometric Analysis (CT‐RSA)

CT scans were performed at two different occasions postoperatively, as mentioned above. Metal Reduction Protocol (MAR) was used on all cases. All CT examinations were performed using a Planmed Verity CBCT (Cone Beam Computed Tomography) scanner (Planmed OY, Helsinki, Finland). A predefined scan protocol was used with the following imaging parameters: tube voltage 90 kV, tube current 6 mA (manual), tube load 36 mAs, pixel size 0.25 mm, slice thickness 0.25 mm, detector coverage 100 mm (isocenter), reconstruction 0.25 mm. All CT‐RSA analyses were conducted by one of the authors (O.S.) using CTMA v23.1 (Sectra, Linköping, Sweden). Tantalum markers which were embedded in the proximal and distal radius osteotomy segments were utilized as reference points. In this paper, we chose to use the tantalum markers as reference points in the analysis to standardize the comparison of the measurements of the two methods as much as possible. Three random CT scans from the cohort were selected and accessed to determine the optimal threshold registration settings for the bone and markers for this patient cohort. The analysis of the CT scans was done stepwise:

1. The two CT datasets of each patient were uploaded into the CT‐RSA software.
2. The same tantalum markers that had been used in marker‐based RSA in the proximal radius fragment were identified as the reference body (Figure 3.1A,B).
3. The software then automatically matched the reference body in the CT‐scan pair as closely as possible. A color‐coded overlay output provided visual aid to help in determining whether the matching process was performed properly or needed correction (Figure 3.1C).
4. The moving body, in this case, the tantalum markers of the distal radius segment, was identified (Figure 3.2A,B). The same tantalum markers were used as in marker‐based RSA.
5. The position of the distal radius segment in the scans was automatically matched as closely as possible to each other. A color‐coded overlay output, as in Step 4, provided visual aid to help determining whether the matching process was performed properly (Figure 3.2C).
6. The geometrical “center of mass” was calculated by the software for each moving segment, by analysing the three‐dimensional position of the markers (Figure 3.3). The coordinate system was defined (Figure 3.4) according to the coordinate system used in RSA for that particular patient. This was done by importing known RSA bead coordinates from RSA into the CT‐RSA software.
7. Numerical data on segment micromotion was obtained in six degrees of freedom (translations along and rotations around *x*, *y*, and *z*‐axis) [19].

**Figure 3:**
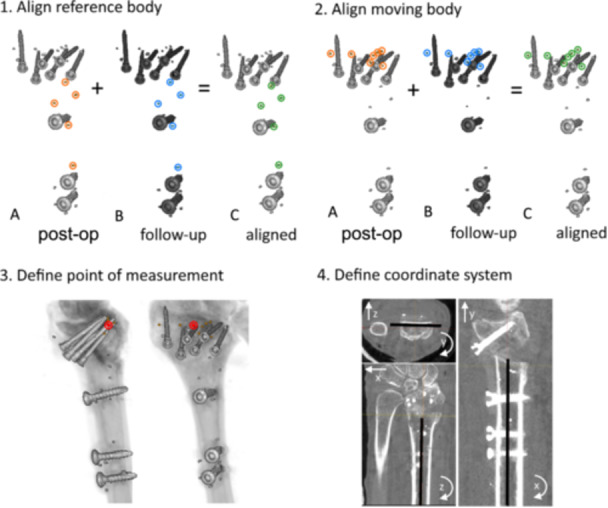
Registration of the tantalum markers of the proximal radius bone segment as reference object on two different CT scans (3.1A and 3.1B) and verification of the overlap process with the help of chromatic overlay (C). Registration of the tantalum markers of the distal radius bone segment as moving body on two different CT scans (2A and 2B) and verification of the overlap process with the help of chromatic overlay (2C). Definitions of the measurement point (3). Definition and adjustment of the coordinate system (4).

### 2.4 Coordinate Systems

The coordinate system utilized in RSA and CT‐RSA is not identical. RSA uses an anatomical fixed coordinate system that is dependent on the calibration cage. On the other hand, CT‐RSA makes use of a standard anatomical DICOM coordinate system. To get comparable coordinate systems, adjustments of the CT coordinate system were performed to match that of the RSA calibration cage.

The marker‐based RSA method in the wrist and hand can give measurements of significant variation depending how the patient's forearm is placed in the calibration cage, previously studied by Jensen et al. [23]. As far as we know, the impact of this on the measurements has not been previously quantified for CT‐RSA. The CT‐RSA method was able to adapt the coordinate system both to the anatomy definition RSA strived for, and to the actual position each patient's forearm had in the RSA measurement. This adaptation made it possible to quantify the impact of the differences between the actual coordinate system used for RSA and the reported coordinate system used in RSA measurements, depicted in the Bland–Altman plots of these two methods of coordinate adjustment (Figure 5).

### 2.5 Radiation Dose

The mean effective radiation dose for the scans used in the CT‐RSA analysis was estimated to be 2 µSv per scan. The corresponding mean effective dose of marker‐based RSA was estimated to be 0.02 μSv per scan. For comparison, one simple set of dental radiographs generates about 5–10 μSv [24].

### 2.6 Statistics

All statistical analysis was performed using IBM SPSS version 27.0.0 software. The level of significance was set to 0.05 for all tests (*α* = 0.05) and all performed tests were two‐tailed. Descriptive statistics were used to calculate the standard deviation (SD) of the differences between the double RSA examinations. The precision of the RSA measurements was defined as “the degree to which repeated measurements under unchanged conditions show the same results and it refers to random errors only” [25]. The formula used to calculate precision was defined as Precision = SD × *t*(*n* − 1) [26], where (SD) corresponds to the standard deviation of the differences calculated between double RSA examinations multiplied by the critical value (*t*) found in the t‐table adjusted for the number of observations minus 1 (*n* − 1). The double marker‐based RSA analysis was done when the osteotomy was deemed to be clinically and radiologically healed. Thus, it was assumed that no true motion of the plate or the distal radius segments could occur between the examinations to calculate precision [26]. The Pythagorean theorem (Total Translation = $\sqrt{X^{2}+\Upsilon^{2}+{Ζ}^{2}}$ [27] was used to calculate the total translation. The simplified Euler's rotation theorem (Total Rotation = $\sqrt{\theta_{x}^{2}+\theta_{y}^{2}+\theta_{z}^{2}}$), which, according to previous research, can be used when micromotions are studied [2, 28], was used to calculate the total rotation. To compare the two methods, Bland–Altman plot analysis and paired analysis were performed. The sample size in this study is relatively small; and thus, normality of the differences in translation and rotation between the RSA and CT‐RSA was examined using a histogram with density curve. It was evaluated graphically that the distribution of the differences for each paired comparison were roughly normal. Paired analysis was performed to compare micromotion measurements of the two methods on all axes. The smallest clinically relevant size of the differences was defined per axis of micromotion as previously stated in the introduction. Due to the relatively small sample of the cohort an agreement analysis between marker‐based RSA and CT‐RSA up to 1 year follow‐up was tested using the Bland–Altman plot analysis. The bias and Limits of Agreement, including the respective 95% confidence intervals, are reported in the plots.

### 2.7 Ethics and Data Sharing

All included patients gave written informed consent to participate in the study. Approval for the study was obtained through the Regional Ethics Review Board in Gothenburg, Sweden, entry number 472‐14. Sharing of this study's research data is available upon request, though personal information of patients cannot be disclosed.

## 3 Results

The precision of the RSA set up in this study is presented in detail in Table 1. For rotations around the *x*‐axis (dorsal/volar tilt), the paired analysis showed a mean difference (95% CI) of −0.06° (−0.67 to 0.55). Correspondingly for translations along the *y*‐axis (radial shortening) the mean difference (95% CI) was −0.04 mm (−0.09 to 0.01), for rotations around the *z*‐axis (radial inclination) 0.21° (−0.06 to 0.48) and for translations along the *x*‐axis (radial shift) −0.07 mm (−0.21 to 0.07). The paired analysis for micromotions on all axes showed that the thresholds of clinical significance are excluded from the difference's 95% confidence interval (Table 2).

**Table 1:** Precision^a^ of marker‐based RSA in measuring distal radius segment migration and rotation based on 19 double examinations.

	RSA	
	SD	Precision
Translations (mm)		
*x*‐axis (medial/lateral)	0.14	0.29
*y*‐axis (proximal/distal)	0.04	0.09
*z*‐axis (anterior/posterior)	0.21	0.44
Total displacement		
Total translation (mm)	0.22	0.46
Total rotation (°)	0.52	1.08
Rotations (°)		
*x*‐axis (anterior/posterior)	0.49	1.03
*y*‐axis (internal/external)	0.40	0.83
*z*‐axis (increased/decreased inclination)	0.40	0.84

^a^Precision is defined as the margin of error that is equal to standard deviation (SD) × critical value from the *t*‐table adjusted for the number of observations minus 1. A 95% significance level was used.

**Table 2:** Paired mean change (mean micromotion difference) for micromotions on all axes for both the CT‐RSA and RSA methods. The mean translation and rotation and the corresponding 95% confidence intervals (CI) are presented on all orthogonal axes.

CT‐RSA–RSA	Mean micromotiondifference	95% CI
Translations (mm)		
*x*‐axis (medial/lateral)	−0.07	−0.21 to 0.07
*y*‐axis (proximal/distal)	−0.04	−0.09 to 0.01
*z*‐axis (anterior/posterior)	0.04	−0.09 to 0.17
Total motion		
Total translation (mm)	0.07	−0.04 to 0.18
Total rotation (°)	0.20	−0.24 to 0.64
Rotations (°)		
*x*‐axis (anterior/posterior)	−0.06	−0.67 to 0.55
*y*‐axis (internal/external)	0.18	−0.18 to 0.54
*z*‐axis (inclination)	0.21	−0.06 to 0.48

**Figure 4:**
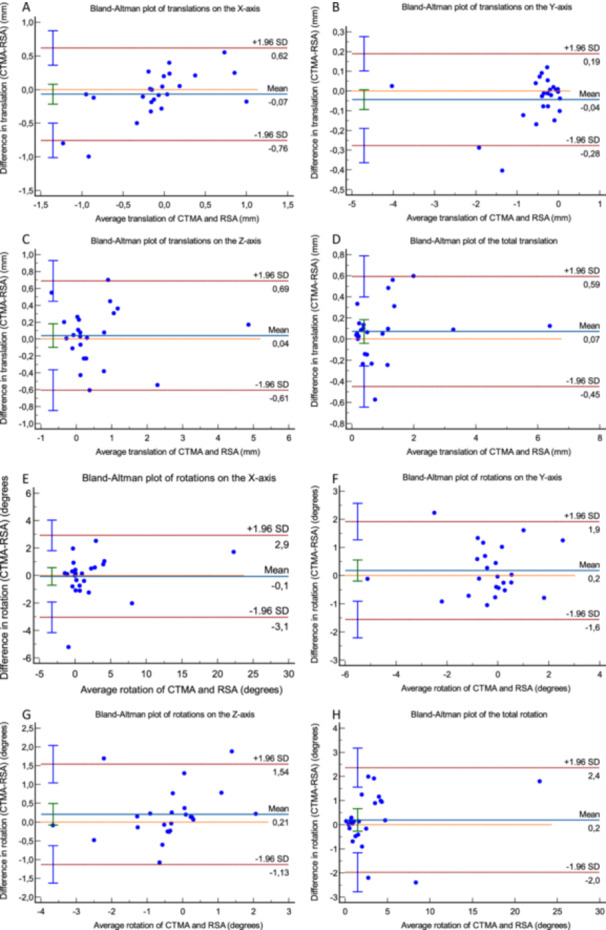
(A–D) Bland–Altman plot for translations on the three orthogonal axes as well as total translation. Limits of Agreement are shown as dotted, red lines with 95% confidence intervals in light blue. Bias is shown as a solid, blue line with the 95% confidence interval in green. The line of equality is shown as a dotted, orange line. (E–H) Bland–Altman plot for rotations around the three orthogonal axes as well as total rotation. Limits of Agreement are shown as dotted, red lines with 95% confidence intervals in light blue. Bias is shown as a solid, blue line with the 95% confidence interval in green. The line of equality is shown as a dotted, orange line.

The Bland–Altman plots (Figure 4E) for rotations around the *x*‐axis (dorsal/volar tilt) shows Limits of Agreement (LoA) being −3.1° (lower) and 2.9° (upper). Correspondingly for translations along the *y*‐axis (radial shortening) the LoA [upper, lower] were [−0.28 mm, 0.19 mm], for rotations around the *z*‐axis (radial inclination) [−1.13°, 1.54°], for translations along the *x*‐axis (radial shift) [−0.76 mm, 0.62 mm]. 95% of all data points in these plots are included within the corresponding LoA. No proportional bias was observed in the plots.

**Figure 5:**
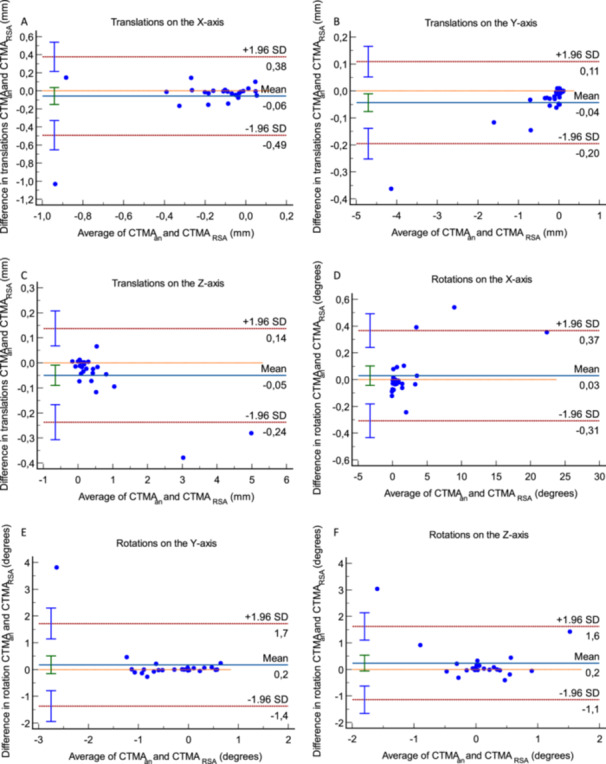
(A–F) Bland–Altman plots of the measurements performed with the CT‐RSA method with coordinate system adjusted to the anatomy definition RSA strived for, versus the actual placement of each patient's arm. Limits of Agreement are shown as dotted, red lines with 95% confidence intervals in light blue. Bias is shown as a solid, blue line with the 95% confidence interval in green. The line of equality is shown as a dotted, orange line. The two outliers on all of the plots represent the same two patients.

The Bland–Altman plots of the discrepancies in measurement using CT‐RSA adjusted to the actual RSA coordinates and CT‐RSA using coordinates adjusted to the radius anatomy definition RSA aimed for (Figure 5A–F) showed that the mean differences are very close to 0 mm, with almost all data points being close to 0 mm and 95% of them within the corresponding LoA. Two outliers were noted, which were the same in all plots.

## 4 Discussion

This is to our knowledge the first study to evaluate the migration pattern of wrist osteotomies using RSA and CT‐RSA in the same patient cohort and one of the few studies to utilize CBCT for the CT‐RSA [29].

There is unfortunately not a clear consensus to which all orthopedic surgeons agree on regarding the amount of motion or micromotion that is acceptable in wrist osteotomies. Thus, to our knowledge, there is no clear guideline or threshold regarding micromotions in the wrist. Previous research has shown that for the definition of an acceptable distal radius reduction, a moderate to high consensus was found among Orthopedic surgeons: a radial inclination of ≥ 15°, a radial height of > 5 mm, and dorsal angulation of < 10° [30]. Other researchers studying functional outcomes after corrective osteotomy for malunion of the distal radius considered that the malunion warranted surgery if there was a dorsal tilt of > 20° with or without one or more of the following parameters: Radial inclination < 10°, radial shift > 5–10 mm and radial shortening > 5 mm [31]. Adapting these threshold values as well as we could as guidelines, we decided to use the lower, stricter values of these parameters as thresholds of acceptable radius displacement. Thus, the limits of acceptable distal radius displacement were set to: dorsal/volar tilt < 10° (rotation in the *x*‐axis), radial shortening < 5 mm (translation in the *y*‐axis), radial inclination ≥ 15° (rotation in the *z*‐axis) and radial shift < 5 mm (translation in the *x*‐axis).

Holm‐Glad et al. [32] have previously studied micromotions in wrist arthroplasties with the help of model‐based RSA on phantoms and patients. In their research precision values of 0.06–0.18 mm for translations and 0.32°–2.18° for rotations are reported for patients [32]. Downing et al. [7], when assessing micromotion in distal radius fractures, found a precision (as 95% error limit) that ranged from 0.06 to 0.13 mm for translations and 0.5°–0.8° for rotations. Thillemann et al. [9] have used the noncommercial CT‐based AutoRSA method to access stability of the distal radioulnar joint. This method had been previously validated with a precision of less than 0.16 mm for translations and 0.71° for rotations [33]. Precision of the marker‐based RSA for distal radius osteotomies in this study varied from 0.09 to 0.44 mm for translations and 0.83°–1.03° for rotations (Table 1).

The Bland–Altman plots of the measurements using CT‐RSA adjusted to the actual RSA coordinates and CT‐RSA using coordinates adjusted to the radius anatomy definition RSA aimed for, show minimal discrepancies. Two outliers were noted. These combined unusually large fragment movement and unusually large malalignment of the coordinate system. However, the LoA in the plots for rotation around the *x*‐ and *z*‐axis, as well as translations along the *y*‐ and *x*‐axis are very narrow from a clinical perspective (Figure 5A–F) with 95% of the measurements being withing the LoA and corresponding 95% CI. We consider, thus, that the malalignment of the patient's forearm in the marker‐based RSA calibration cage was overall a negligible source of error for the RSA quantifications in this study. The highest values of precision for RSA in the current paper were 0.44 mm for translations and 1.03° for rotations. The Bland–Altman plots for the primary outcome measure, namely rotations around the *x*‐axis, show LoA that are above those limits (LoA −3.1, 2.9), but still within the threshold for what we defined as clinically important in the current paper for rotations on the *x*‐axis. The same goes for rotations around the *z*‐axis. The Bland–Altman plots for translations on the *y*‐axis show LoA that are within those limits, while for translations in the *x*‐axis slightly above these limits (LoA −0.76, 0.62) but still within the limits of what is considered clinically significant according to our thresholds. In the current study the mean values of the difference in translation and rotation and the values of LoA, as well as the corresponding 95% CI were below the values that in our consideration and experience are clinically significant. 95% of the data points in the corresponding graphs were within the LoA. No proportional bias was noted. Furthermore, the paired analysis for micromotions on all axes indicates a comparability of the two methods as all clinically relevant differences are excluded from the 95% CI. Our data indicate, thus, that the discrepancies of measurements between the two methods are not clinically significant.

The CT‐RSA method does not necessarily require tantalum markers for the analysis, as bone or implant surfaces are used as reference and moving objects in the analysis instead. Methodological problems, thus, regarding occluded markers and patient exclusion due to improper marker placement or very high CN could be eliminated, when this technique is used. These facts could also enable inclusion of patients even retrospectively after the surgery is performed. In the current paper we chose to use markers also in the CT‐RSA analysis to get the clearest possible comparison of the measurements of the two methods when using the exact same rigid bodies, measurement points, and coordinate system.

An advantage of CT‐RSA is the wide availability of CT scanners, which enables the initial and follow‐up examinations to be conducted in smaller institutions with standard CT scanners without the need to access an RSA laboratory. This is a significant aspect of, among others, implant introduction studies [34]. In addition to this, CT‐RSA as an analyzing tool makes measuring implant migration easier by using predetermined CT‐scan protocols.

A limitation of CT‐RSA is that there is yet a lack of a quantitative measure of the correctness of the measurements such as the CN and mean error (ME) as in RSA. This is an aspect that has been discussed also in previous studies [19]. Color‐coded feedback, provided by the CT‐RSA software, visually aids the analyst to evaluate the correctness of the analysis [18]. That said, the assessment is relied on the analyst's or user's experience with the software. Despite that, according to previous research even a relatively inexperienced CT‐RSA user can produce reliable measurements with very high inter‐ and intra‐observer repeatability, as high as 0.88–0.99 and 0.90–0.99, respectively, as reported on previous research by Sandberg et al. on hips [35].

Another limitation of this study was that we utilized tantalum markers also in the CT‐RSA analysis. As stated above, that was done with the intention of comparing the marker‐based RSA and CT‐RSA as similarly as possible. In future studies that do not have focus in comparing the two methods, rather studying micromotions in wrist osteotomies, the use of CT‐RSA does not dictate the presence of markers.

In this study interobserver repeatability was not confirmed, because one analyst (B.S.) performed all the RSA measurements, and another analyst (O.S.) all of the CT‐RSA measurements. Our suggestion is that in future studies interobserver repeatability should also be examined.

As the study, patients were drawn from, was designed before the availability of CT‐RSA, we did not include double CT examinations and therefore cannot include precision data for CT‐RSA. We recommend that future RSA and CT‐RSA comparison studies also include that in the analysis.

In the current study, micromotions of distal radius osteotomies were examined. It is, therefore, challenging to extrapolate the results of this study to other joints or implement them on wrist implants (such as wrist arthroplasties). Many parameters should be considered such as metal artefacts, radiation doses, implant design, and follow‐up times. For wrist osteotomies, follow‐up of micromotions after 1 year may not be useful, as complication‐free osteotomies are surely healed within 1 year. Should one, though, conduct implant studies in the wrist, where migration under longer periods of time is possible (such as in wrist arthroplasties), longer time intervals and additional follow‐up occasions are advisable.

## 5 Conclusion

In conclusion, the reliability of the CT‐RSA method, according to our data, is comparable to that of marker‐based RSA in measuring micromotions after wrist osteotomy, as the differences between the methods are not clinically significant.

## Author Contributions

Michael Ullman conceptualized the study and operated all patients. All authors were involved in the study design. Bita Shareghi and Vasileios Angelomenos retrieved and prepared the data. Bita Shareghi and Olof Sandberg performed the RSA and CT‐RSA analyses. Vasileios Angelomenos performed the statistical and critical analyses and wrote the manuscript. All authors contributed with notable critical appraisal of the text and approved the final version.

## Conflicts of Interest

Olof Sandberg works as an engineer and researcher at Sectra, the company that owns CTMA, the CT‐RSA analysis software that was used in this study, which could imply conflict of interest, but he had no part in the clinical interpretation of the results. The other authors declare no conflicts of interest.

## Supporting information

Supporting information.
